# A unique case of Miller Fisher-Guillain-Barré overlap syndrome in a liver transplant recipient

**DOI:** 10.1007/s13365-021-01015-6

**Published:** 2021-09-22

**Authors:** Claudia Ramirez-Sanchez, Rehan Syed, Angela Meier, Jamie Nicole LaBuzetta, Diana J. Hylton, Mahnaz Taremi

**Affiliations:** 1 Department of Infectious Diseases and Global Public Health, University of California San Diego, La Jolla, San Diego, CA USA; 2 Department of Anesthesiology, University of California San Diego, La Jolla, San Diego, CA USA; 3 Department of Neurosciences, Division of Neurocritical Care, University of California San Diego, La Jolla, San Diego, CA USA

**Keywords:** Descending paralysis, Guillain-Barré Syndrome, Miller Fisher syndrome, Liver transplantation, COVID-19

## Abstract

Guillain-Barré syndrome (GBS) is an ascending demyelinating polyneuropathy often associated with recent infection. Miller Fisher syndrome represents a variant with predominant facial and cranial nerve involvement, although Miller Fisher and Guillain-Barré overlap syndromes can occur. Guillain-Barré spectrum syndromes have been thought to be rare among solid organ transplant recipients. We describe an immunocompromised patient with a liver transplant who presented with ophthalmoplegia and bulbar deficits. His symptoms rapidly progressed to a state of descending paralysis involving the diaphragm; he then developed acute respiratory failure and eventually developed quadriparesis. Electromyography and a nerve conduction study demonstrated a severe sensorimotor axonal polyneuropathy consistent with Miller Fisher variant Guillain-Barré syndrome. Despite several negative nasopharyngeal swabs for COVID-19 polymerase chain reaction, a serology for SARS-CoV-2 IgG was positive. He was diagnosed with Miller Fisher-Guillain-Barré overlap syndrome with rapid recovery following treatment with plasma exchange. Although Guillain-Barré is a rare complication in solid organ transplant recipients, this case highlights the importance of rapid diagnosis and treatment of neurologic complications in transplant patients. Furthermore, it demonstrates a possible case of neurological complications from COVID-19 infection.

## Background

Traditionally, Guillain-Barré Syndrome (GBS) has been thought to be less common among solid organ transplant recipients (El-Sabrout et al. 2001). Herein, we describe an immunocompromised patient after liver transplantation who presented with symptoms of acute progressive descending weakness initially concerning for botulism, but ultimately consistent with variant GBS. Additionally, we postulate that COVID-19 infection may have been a trigger for his development of GBS given laboratory findings suggestive of recent infection, and in the context of case reports of Miller Fisher syndrome among patients with COVID-19 infection (Dimachkie and Barohn 2013).

## Case presentation

A 55-year-old man with a history of orthotopic liver transplantation for hepatitis C virus cirrhosis 7 months prior presented to a local emergency department after noticing slurring his words and a heavy tongue at home. His other medical history included type II diabetes mellitus and end-stage renal disease requiring intermittent hemodialysis. He had been taking cyclosporin as a single immunosuppression medication, with his mycophenolate mofetil held temporarily the following bacteremia with *Pseudomonas aeruginosa* a month prior. The initial stroke workup included computed tomography (CT) of the head and CT angiography of the head and neck, which did not reveal any acute abnormalities. He progressively developed respiratory failure, hypoxemia, and hypercapnia in the setting of bulbar weakness. He was intubated and was immediately transferred to our hospital. Upon arrival to our intensive care unit, he remained alert and interactive, answering yes or no questions with a flickering of his right foot. His vital signs included a blood pressure of 155/92 mm/Hg, a pulse rate of 88 beats per minute, and a temperature of 98.7 °F. Neurologic examination was significant for bilateral ptosis, weakness of cranial nerves III, IV, VI, and VII, and decreased motor strength in the proximal and distal muscles of the bilateral upper limbs. Laboratory data upon admission were notable for a white blood cell count of 7900 cells per mm (Manganotti et al. 2020), BUN 53 mg/dL (18.9 mmol/L), and CRP of 8.67 mg/dL (825.7 nmol/L). Cyclosporin blood level was not toxic at 40 ng/mL (33.28 nmol/L), and his most recent dialysis session had been 3 days prior to admission. His other liver and renal function tests were unremarkable. The patient’s weakness worsened rapidly over the next 24 h, progressing to flaccid quadriparesis with the absence of all deep tendon reflexes on examination. He received a dose of heptavalent anti-toxin for the concern of botulism with no appreciable clinical response. He was also incidentally noted to have nasal myiasis, for which he underwent endoscopic removal of larvae and treatment with ivermectin.

Our patient did not demonstrate symptoms of acute COVID-19 viral syndrome on admission and a CT scan of the chest showed no acute abnormalities. A rapid nasal swab for COVID-19 polymerase chain reaction (PCR) was negative. A subsequent nasopharyngeal swab for COVID-19 PCR performed over this hospitalization was also negative. He had been admitted 2 weeks prior to this admission with sudden onset of dyspnea and hypoxemia, requiring supplemental oxygenation. At that time, his symptoms were thought to be secondary to hypervolemia, as his oxygenation improved though did not resolve, after receiving hemodialysis. During that admission, his chest imaging (chest X-ray and CT chest) showed increased airspace opacities involving bilateral lung fields as well as small pleural effusions. His rapid nasal swab PCR for COVID-19 was negative at that time and so was his respiratory panel, including influenza and *Mycoplasma pneumoniae* PCR. In the context of recent hypoxemia, and concern for COVID-19 infection despite the negative PCR result, he was tested for SARS-CoV-2 antibodies during the current admission which was positive for IgG. As part of his evaluation, he also underwent a lumbar puncture. Cerebrospinal fluid (CSF) analysis demonstrated albuminocytologic dissociation with high protein at 110 mg/dL and cell counts notable for only one WBC and three red blood cells. Glucose was 70 mg/dL. CSF cultures and a PCR panel for possible organisms such as bacteria, herpes simplex virus, and cytomegalovirus (CMV) were negative. A SARS-CoV-2 PCR run on CSF fluid was negative. Other studies including Lyme disease serology, human immunodeficiency virus (HIV), mycoplasma IgM, and syphilis testing were negative.

Magnetic resonance imaging (MRI) of the brain and cervical spine did not show any acute findings or enhancing lesions. Nerve conduction studies (NCS) and electromyography (EMG) demonstrated a severe, length-dependent, and symmetrical sensorimotor axonal polyneuropathy, but no signs of presynaptic junction disorder (Tables 1 and 2). These findings were uncharacteristic of botulism and more consistent with GBS.

**Table 1:** Nerve conduction studies

Nerve stimulated	Stimulation site	Record site	Latency (ms)	Amplitude (µV)	Velocity (m/s)
Right radial (s)	Forearm	Snuffbox	2.7	8.3	48
Right radial (s)	Wrist	Digit five	2.4	6.7	46
Right median (m)	Wrist	Abductor pollicis brevis	NR	NR	NR
Left peroneal (m)	Ankle	Extensor digitorum brevis	NR	NR	NR
Left tibial (m)	Ankle	Abductor hallucis brevis	NR	NR	NR
Left ulnar (m)	Wrist	Abductor digiti minimi	NR	NR	NR
Right ulnar (m)	Wrist	Abductor digiti minimi	NR	NR	NR

Right radial and ulnar sensory nerve conduction were moderately reduced in amplitude. Left ulnar, superficial peroneal, and sural sensory nerve conductions were absent (not shown). Right median, ulnar motor conductions were absent. Left ulnar, peroneal, and tibial motor conductions were absent. Left and right blink reflexes showed no response bilaterally (not shown)

*S* antidromic sensory, *NR* no response

**Table 2:** Needle electromyography results

Muscle	Insertional activity	Spontaneous activity			Voluntary activity			
		PSW	Fibrillation	Fasciculation	Amplitude	Duration	Polyphasic	Recruitment
Left deltoid	Normal	Normal	Normal	Normal	1-	1-	Normal	Rapid
Left biceps	Normal	Normal	Normal	Normal	1-	1-	Normal	Rapid
Left triceps	Normal	Normal	Normal	Normal				None
Left pronator teres	Normal	Normal	Normal	Normal	1-	1-	Normal	Rapid
Left first dorsal interosseous	Normal	Normal	Normal	Normal	1-	1-	Normal	Rapid

Needle electromyography of selected muscles of the left upper extremity showed a myopathic pattern of rapid recruitment of small amplitude, short-duration motor unit potentials in the deltoid, biceps, pronator teres, and first dorsal interosseous muscles. The triceps demonstrated no units recruited during volitional activation

*PSW* positive sharp waves

Despite negative testing for anti-ganglioside antibodies—including anti-GM1, anti-GD1b, and anti-GQ1b—MFS-GBS overlap was suspected given the combination of CSF findings, EMG/NCS results, clinical signs, and course more consistent with autoimmune neuritis. Plasma exchange therapy was initiated, and he showed marked clinical improvement after each session. He underwent a total of seven sessions. After his final session of plasma exchange, he was successfully weaned from mechanical ventilation. He was discharged after spending 22 days in the hospital. On the day of discharge, his motor strength was 4/5 bilaterally in all limbs, with significant improvement in his multiple cranial nerve palsies.

## Discussion and conclusions

The diagnosis of MFS-GBS overlap was made based on clinical features including acute onset of ophthalmoplegia and areflexia followed by subsequent progressive descending flaccid weakness, laboratory findings such as CSF albuminocytologic dissociation, and results from EMG/NCS. While anti-GQ1b was not detected, there have been prior cases of MFS in which this antibody test has been negative (Manganotti et al. 2020; Wattanasit and Sathirapanya 2020). Additionally, our patient experienced impressive rapid recovery of his neurological symptoms after initiation of plasma exchange, which is consistent with other reports that have found plasma exchange therapy to be effective in the treatment of GBS and its variants (Berg et al. 2014; Zito et al. 2020).

GBS variant demyelinating disease can mimic other neurologic disorders such as myasthenia gravis and botulism, which were also considered (Anthony et al. 2012; Legast et al. 2017). Our patient initially received heptavalent anti-toxin with a concern for botulism with suspicion prompted in the setting of extensive nasopharyngeal myiasis, several cranial nerve palsies, and descending motor impairment. However, EMG/NCS studies were felt to be incompatible with this diagnosis. The NCS were abnormal with evidence of a severe, length-dependent, sensorimotor polyneuropathy with axonal features. The complete absence of motor conductions was most likely due to severe blocking, as seen in demyelinating diseases like GBS. In contrast, botulism was not supported by the study since all motor NCS were completely absent; botulism classically produces low amplitude motor conductions that increase with short exercise testing (Oh 1977). Bilateral involvement of the trigeminal nerve also pointed towards a diffuse neuropathy involving the sensory nerves, such as GBS. In addition, there was a non-irritable myopathy, most likely reflecting critical illness myopathy (Latronico and Bolton 2011).

In the presented case, the trigger for MFS-GBS overlap disease was unclear. GBS is broadly thought to be triggered by an immune response and is considered to be a relatively rare phenomenon in immunosuppressed patients, including solid organ transplant recipients. In cases where it has been identified, it has been most associated with a respiratory or gastrointestinal infection, acute CMV infection, influenza vaccination, calcineurin-inhibitor neurotoxicity, and allograft rejection (El-Sabrout et al. 2001; Lo Re et al. 2018). The patient had experienced *Pseudomonas* bacteremia 1 month prior to his onset of symptoms; however, given the organism and duration since infection, this was felt to be less likely to be the trigger. As described, he experienced respiratory symptoms 2 weeks prior to the onset of his symptoms, which was felt to be the possible triggering event. However, his workup at the time for infectious causes, including viral, was negative except for his SARS-CoV-2 IgG test, which was positive.

Given multiple negative nasal COVID-19 PCR tests, it is not possible to determine definitively if COVID-19 infection was present. Furthermore, given the in-house development of the SARS-CoV-2 IgG test used at the time, population specificity is not readily available. However, of note, studies of other similar assays at the time showed specificities in the 96–99% range (Lisboa Bastos et al. 2020). The duration of persistence of IgG antibodies to SARS-CoV-2 varies; however, case reports suggest GBS may develop 1–4 weeks after infection with the SARS-CoV-2 virus which would be consistent with the timing of developing these antibodies (Hirayama et al. 2020). Given the onset of the pandemic in the USA, 4 months prior to his presentation, it is unlikely that his positive test reflected remote infection or vaccination, and in the context of an extensive workup without implication of an alternative trigger, recent infection with the virus was felt to be plausible. Therefore, in the absence of a clear alternative cause, it is plausible but not definitive that COVID-19 infection may have been the trigger for our patient’s development of GBS. Indeed, there have been few cases of GBS reported in kidney transplant recipients with a COVID-19 infection, but none in liver transplantation (Rajdev et al. 2020; Yaqoob et al. 2020). There has been postulation that cross-reactivity between peripheral nerve glycolipids and epitopes within gangliosides involved in SARS-CoV-2 viral binding may underlie the pathogenesis of these syndromes (Dalakas 2020). This would be similar to other infectious syndromes associated with GBS, in which molecular mimicry and autoimmune response are implicated (Hughes et al. 1999).

To date, few cases of GBS have been reported in immunocompromised patients such as those with a history of solid organ transplant. In this report, we describe a rare variant of GBS in a patient with a history of orthotopic liver transplantation. Furthermore, we raise the possibility that a recent COVID-19 infection may have played a role in the development of this GBS variant. The clinical syndrome was largely characterized by progressive descending paralysis and was successfully treated with plasma exchange treatments. This case highlights the importance of a prompt evaluation of all immunocompromised patients presenting with neurological symptoms suggestive of GBS spectrum, given the high mortality rate and related disabling neurological deficits associated with delays in diagnosis or treatment.

## Data Availability

Not applicable

## Abbreviations

CMV: Cytomegalovirus
COVID-19: Coronavirus disease of 2019
CSF: Cerebrospinal fluid
CT: Computed tomography
EBV: Epstein-Barr virus
EMG: Electromyography
GBS: Guillain-Barré syndrome
HIV: Human immunodeficiency virus
MFS: Miller Fisher syndrome
MRI: Magnetic resonance imaging
NCS: Nerve conduction studies
PCR: Polymerase chain reaction
SARS-CoV-2: Severe acute respiratory syndrome-coronavirus-2
